# Disorder‐Induced Extremely Low Thermal Conductivity of Graphite Fluoride

**DOI:** 10.1002/advs.202518438

**Published:** 2026-05-08

**Authors:** Wonsik Lee, Donghoon Moon, Ziyan Qian, Jinwoo Kim, Changheon Kim, Yeojin Lee, Hyobin Yoo, Richard B. Wilson, Qiye Zheng, Gwan‐Hyoung Lee, Hyejin Jang

**Affiliations:** ^1^ Department of Materials Science and Engineering Seoul National University Seoul Republic of Korea; ^2^ Department of Mechanical and Aerospace Engineering The Hong Kong University of Science and Technology Hong Kong SAR China; ^3^ Research Institute of Advanced Materials Seoul National University Seoul Republic of Korea; ^4^ Department of Materials Science and Engineering & Mechanical Engineering University of California Riverside Riverside California USA

**Keywords:** anisotropy, disorder, graphite fluoride, thermal conductivity, time‐domain thermoreflectance

## Abstract

Graphite fluoride represents an important class of functionalized graphene‐based materials. While theoretical studies have extensively examined the functionalization effects on graphene's thermal conductivity, experimental establishment of fluorination effects on thermal transport remains limited, especially regarding the through‐plane thermal conductivity of the layered structures. We determine the through‐plane and in‐plane thermal conductivity of mechanically exfoliated graphite fluorides using co‐aligned and beam‐offset time‐domain thermoreflectance, cross‐validated with frequency‐domain thermoreflectance. Graphite fluorides exhibit the exceptionally low through‐plane thermal conductivity of < 0.030 W m^−1^ K^−1^ for thicknesses of 53–243 nm at room temperature – the lowest among fully dense solids. The in‐plane thermal conductivity is measured as 4.2–5.6 W m^−1^ K^−1^ for a 178 nm‐thick flake, resulting in an anisotropy ratio of thermal conductivity exceeding 100. The record‐low through‐plane thermal conductivity stems from widely distributed interlayer spacing with configurational and stacking disorders as well as mass and rotational disorders, while reduced in‐plane thermal conductivity results from nm‐sized fluorinated grains with configurational disorder. We further investigate the effect of fluorine incorporation on surfaces, revealing that surface fluorination minimally impacts interfacial thermal conductance. Our results demonstrate how structural disorder creates unprecedented lower bounds for thermal conductivity in layered materials modified by functional groups.

## Graphical abstract

This article investigates the effect of fluorination on the thermal conductivity of graphite fluoride, demonstrating the relationship between structural modification and thermal conductivity in an extreme case. The record‐low through‐plane thermal conductivity measured in exfoliated graphite fluoride flakes was found to correlate with the wide variation in interlayer spacing as well as the mass and rotational disorders.

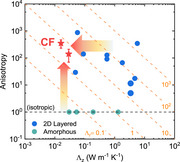

## 1 Introduction

2D layered materials exhibit intrinsic anisotropy in thermal conductivity, characterized by significant differences between the in‐plane and through‐plane directions. This anisotropy arises from their layered structures, where weak van der Waals (vdW) bonding exists between layers, while strong covalent bonding dominates within the layers. The degree of anisotropy is further expanded by the influence of the constituent atoms and their arrangement. Owing to these characteristics, the thermal conductivity of 2D layered materials exhibits an exceptionally wide range of thermal conductivity spanning over five orders of magnitude within this material group. At one extreme, graphene shows ultrahigh in‐plane thermal conductivity, i.e., 2000–5000 W m^−1^ K^−1^ at room temperature [1, 2, 3, 4, 5, 6]. At the other extreme, turbostratic WSe_2_ shows the record‐low thermal conductivity in the through‐plane direction, i.e., 0.048 W m^−1^ K^−1^ at room temperature, due to the rotational disorder on the planes [7]. This remarkable variability highlights the diverse thermal transport properties of 2D layered materials driven by their atomic and structural characteristics.

Recent research has successfully tailored the thermal transport properties of 2D layered materials beyond their intrinsic characteristics through various modifications. The strategies include the chemisorption of functional groups [8, 9], intercalation [10, 11, 12], and the construction of a moiré superlattice [13, 14, 15] or vdW heterostructures [16]. Among them, the functionalization of graphene, such as hydrogenation, fluorination, and oxidation, has gained significant attention for its ability to tune thermal transport properties along with electrical and mechanical properties [17, 18, 19, 20]. We note that hydrogen and fluorine can fully functionalize graphene, forming C_1_H_1_ (graphane) and C_1_F_1_ (graphene fluoride) stoichiometry, respectively, while oxygenated functional groups have limited coverages [21].

Functionalization of graphene modifies the one‐atom‐thick, planar crystal structure of graphene, thus significantly altering its intrinsic properties. The functional groups form chemical bonds with carbon atoms and transform carbon hybridization from sp^2^ to sp^3^. This change in hybridization introduces an electronic band gap to semimetallic graphene [22, 23, 24]. The degree of sp^3^ hybridization, controlled by the coverage of the functional groups, can continuously tune the band gap, as demonstrated in graphene oxide (GO) or reduced graphene oxide (rGO), where band gaps range from near 0–3 eV [25, 26, 27], and in hydrogenated graphene, where the band gap reaches up to 4.66 eV [28].

While functionalization offers significant tunability to the electrical properties of graphene, it consistently reduces graphene's ultrahigh in‐plane thermal conductivity to varying degrees. As functional groups typically attach above or below the graphene layer, these chemical bonds distort the atomically planar structure of graphene into buckled structures. The breakdown of in‐plane reflection symmetry drastically lowers the thermal conductivity, and the specific structural modifications further impede the thermal transport properties. Graphane, fully hydrogenated graphene (C_1_H_1_), theoretically demonstrates about 50% reduction in thermal conductivity compared to graphene due to the reduced contribution of heat‐carrying acoustic phonons [5, 29, 30]. Increasing the atomic mass of the functional group to 30 amu (C─^30^H) further suppressed the thermal conductivity to about 235 W m^−1^ K^−1^, less than 10% of pristine graphene [30]. The configuration of functional groups provides an additional tunability of thermal conductivity [20, 31, 32], with graphane showing up to 65% decrease in thermal conductivity of graphene [32].

While the deviation of the thermal conductivity of functionalized graphene from that of pristine graphene has been extensively studied through theoretical calculations, these studies are often limited to idealized monolayer structures. Examples include graphane and graphene fluoride with coverage up to 100% functionalization [20, 33, 34, 35], as well as oxygenated graphene with various bonding coordination and limited coverage [17, 21, 36]. However, the understanding of multilayer structures remains lacking, possibly due to the calculation complexity of simulating interlayer interactions. In addition, real functionalized samples exhibit various defects in their microstructures, such as point defects, grain boundaries, and pores, and may even form agglomerates. This is most noticeable in GO and rGO [37], whose measured thermal conductivities are predominantly controlled by the grain size and porosity rather than hybridization of carbon [38]. For example, the fabricated GO and rGO films exhibit a wide range of thermal conductivity, i.e., 2–30 and 150–1400 W m^−1^ K^−1^ for GO and rGO, respectively, depending on the fabrication process [38, 39, 40, 41]. Since theoretical studies struggle to account for the multilayered and defective nature of real samples, comprehensive experimental investigations are essential to establish the relationship between the thermal and structural properties of these materials and ultimately to bridge the gap between theoretical predictions and practical performance.

Although graphene fluoride and graphite fluoride are also expected to exhibit lower thermal conductivity than graphene, they have gained interest in thermal management applications requiring electrical insulation, despite limited understanding of their thermal transport properties. In fully fluorinated graphene, i.e., C_1_F_1_, carbon atoms completely transform to sp^3^ hybridization [42], creating a wide band gap of about 3.8 eV [24, 43, 44], thus suitable for applications where a large breakdown voltage is needed [43, 45]. Several studies have already demonstrated graphite fluoride as a thermally conductive filler in thermal interface materials [46, 47, 48, 49]. For instance, one study presented 70% enhancement in the thermal conductivity of epoxy at a 1 wt.% of graphite fluoride fillers [48], even a 50‐fold increase in that of epoxy at an 8 vol.% through forming a compressed network of graphite fluoride for a heat pathway [46].

The crystal structures of graphene fluoride and graphite fluoride have been well‐defined, directly influencing their thermal transport properties. Among various possible stoichiometric configurations, the chair configuration is most stable, with fluorine atoms alternately bonded above and below the carbon layer. This arrangement causes the carbon layer to buckle and transforms its space group from P6/mmm of graphene to a less symmetric P‐3m1 [33], with bond angles of *θ*_CCF_≈ 108° and *θ*_CCC_≈ 111° [23, 50]. The in‐plane lattice constant (*a*) of graphite fluorides is in the range of 2.58–2.62 Å [50, 51, 52, 53], approximately 5% larger than that of graphene (2.46 Å) [54]. Similarly, the in‐plane C─C bond length increases by 10%, i.e., 1.577–1.579 Å, compared to graphene's 1.425 Å [23, 33]. In the through‐plane direction, graphite fluoride has a C─F bond length of 1.37–1.38 Å [23, 33], while the ideal interlayer spacing in chair configurations ranges from 5.7–6 Å [50, 52, 53], twice that of graphite (3.34 Å) [55]. Graphene fluoride has three other fundamental configurations, i.e., boat, zigzag, and armchair [23]. While the chair configuration is known to be the most energetically stable, the energy differences among these configurations are remarkably small. For instance, the formation energy difference between the chair and zigzag configurations is only about 0.036 eV per atom, and other configurations exhibit differences of less than 0.1 eV per atom [23]. In the case of stacking sequences of graphite fluoride, AA, AB, ABC, and AA’ stacking of chair configurations display nearly negligible formation energy differences, with variations of up to only 0.02 eV per atom [52].

Aligning with the well‐defined ideal crystal structures, theoretical predictions consistently show reduced in‐plane thermal conductivity for graphene fluoride. The increased in‐plane C─C bond length of graphene fluoride indicates a weaker in‐plane bond strength compared to graphene [33], as the in‐plane elastic modulus of graphene fluoride is 30% reduced [23]. Those weakened in‐plane covalent bonds suggest reduced thermal conductivity in graphene fluoride, with the loss of in‐plane reflection symmetry serving as another critical factor in this thermal transport reduction. Indeed, first‐principles calculations suggest that graphene fluoride exhibits in‐plane thermal conductivity of 145 W m^−1^ K^−1^ at room temperature, approximately 5% of that of graphene [33, 35, 56].

Until now, experimental studies on the thermal conductivity of graphite fluoride are still limited, which strongly depends on the fabrication methods, making comparison with theoretical predictions difficult. The in‐plane thermal conductivity of graphene fluorinated by Xenon disulfide (XeF_2_) gas was measured as about 80 W m^−1^ K^−1^ at 320 K [57], while graphite fluoride films fabricated by vacuum filtration show a wide range, 60–242 W m^−1^ K^−1^ at room temperature [58, 59]. These vacuum‐filtrated films likely do not represent intrinsic properties due to functional group contamination, graphite phase inclusions, or fabrication‐induced defects. Furthermore, the through‐plane thermal conductivity of graphene fluoride remains largely unexplored, although the fluorination has been shown to weaken the interlayer vdW interactions compared to graphite [52]. We also note that fluorine configuration, stacking sequence, and microstructures may deviate significantly from the ideal cases depending on fluorination conditions, potentially altering the lattice properties such as in‐plane bond strength and interlayer interactions [23, 52]. Therefore, similar to rGO and GO, it is crucial to experimentally elucidate the thermal conductivity of graphite fluoride, accompanied by careful structural characterization.

Herein, we experimentally determined the anisotropic thermal conductivity of graphite fluoride (CF) in the form of exfoliated flakes. We carefully characterized the composition and structures of CF flakes by atomic force microscopy (AFM), scanning electron microscopy (SEM) with energy dispersive X‐ray spectroscopy (EDS), X‐ray photoelectron spectroscopy (XPS), transmission electron microscopy (TEM) with electron energy loss spectroscopy (EELS), and measured their thermal conductivity by using time‐domain thermoreflectance (TDTR). Surprisingly, our mechanically exfoliated CF flakes exhibit the lowest thermal conductivity among fully dense materials, i.e., < 0.030 W m^−1^ K^−1^ at room temperature for the thicknesses of 53–243 nm. We also measured the in‐plane thermal conductivity of a 178 nm‐thick CF flake as 4.2–5.6 W m^−1^ K^−1^, which exhibits the anisotropy ratio in thermal conductivity exceeding 100. We further distinguished the effects of fluorination at the surface of CF flakes and within the flakes on the measured thermal conductivity. We believe this work demonstrates that extreme heat insulation can be implemented in disordered and anisotropic materials.

## 2 Results and Discussion

### 2.1 Characterization of Mechanically Exfoliated Graphite Fluoride (CF) Flakes

**FIGURE 1:**
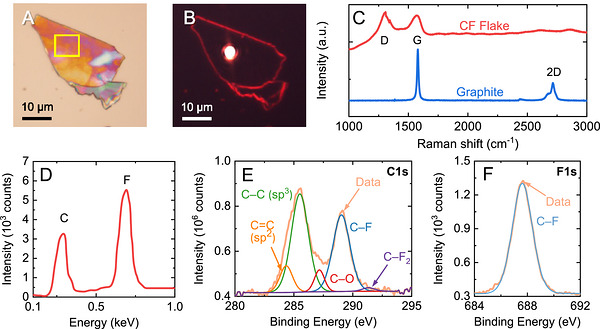
Characterization of exfoliated graphite fluoride (CF). (A) Bright‐ and (B) dark‐field optical micrographs of a 243 nm‐thick CF flake exfoliated on a Si substrate. The yellow box in Figure 1A indicates the area measured by SEM‐EDS. The bright circle in Figure 1B is a probe beam with a 1/e^2^ radius of 1.2 µm. (C) Raman, (D) SEM‐EDS spectra, and (E) C1s and (F) F1s XPS spectra of the CF flake shown in Figure 1A.

Graphite fluoride (CF) flakes were mechanically exfoliated from carbon monofluoride flakes (ACS Materials) onto Si (1 0 0) with a native silicon oxide layer and 500 nm‐thick SiO_2_/Si (1 0 0) substrates. Figure 1A,B presents the bright‐ and dark‐field optical images of a representative 243 nm‐thick CF flake exfoliated on the Si substrate, respectively. The flake thicknesses were determined using AFM (Figure S1). The X‐ray diffraction spectrum of the parent material, carbon monofluoride flakes (Figure S2), revealed that the peaks at 2θ of 13.3° and 40.8° are consistent with prior measurements of graphite fluoride [60, 61]. However, the sharp peaks at 24.6° and 54.6° indicate the additional presence of pure graphite in the parent material, suggesting that both graphite fluoride and graphite could be simultaneously exfoliated onto the substrate.

Raman spectroscopy was performed on the CF flakes to determine the degree of fluorination and compare the spectra with that of pure graphite. Figure 1C shows that the Raman spectra of the 243 nm‐thick flake in Figure 1A and graphite are consistent with previous studies [43, 62, 63]. The exfoliated graphite exhibits high purity, evidenced by the absence of a disorder (*D*) band near 1300 cm^−1^. The *2D* (or *G’*) band of graphite near 2700 cm^−1^ corresponds to the overtone process of the *D* band, which appears for the sample without any defects or disorders [64]. In contrast, CF exhibits a prominent *D* band near 1300 cm^−1^ and lacks a *2D* band, indicating extensive fluorination and the consequent disruption of sp^2^ bonding [65]. The intensity ratio of *D* and *G* peaks (*I_D_*/*I_G_*) of the CF flake is approximately 1.07, further confirming the extensive degree of fluorination [66]. The presence of distinct *D* and *G* peaks of CF suggests a crystalline rather than complete amorphous structure [62, 67].

For the elemental analysis of CF flakes, we performed SEM‐EDS and XPS. Figure 1D presents the SEM‐EDS spectrum, providing semi‐quantitative elemental analysis and revealing the atomic ratio of carbon to fluorine in the sample, i.e., CF_x_ where x = 0.78–1.02. SEM‐EDS allowed for analysis of the entire flake volume, as its penetration depth of several microns exceeds the flake thickness, while XPS is more sensitive to the near‐surface composition, i.e., less than 10 nm from the surface [68]. The XPS of the flake in Figure 1A showed a double‐peaked C1s spectrum between the binding energies of 284 and 290 eV (Figure 1E), and a single peak for F1s at 687.6 eV (Figure 1F), in accordance with the prior studies [58, 69, 70, 71, 72]. Deconvolution of the C1s spectrum revealed a predominance of sp^3^ carbon bonding, which is located at ∼285.5 eV [73]. While previous XPS studies on nearly fully fluorinated graphene (or graphite) reported 3–5 times higher intensity of C─F peak [58, 66, 73, 74] (∼289 eV) than that of C─C peak (285 eV), our sample exhibits comparable intensities of C─C (285.5 eV) and C─F (∼ 289.0 eV) peaks, suggesting incomplete fluorine coverage on the flake surface. XPS analysis also determines the chemical composition of CF near the surface by comparing the sp^2^ and sp^3^ bonding ratios of carbon, assuming complete formation of sp^3^ bonds between carbon and fluorine. This analysis reveals that the CF flake in Figure 1 has a chemical composition of CF_0.75_, which differs by less than 10% from the SEM‐EDS results.

### 2.2 Sound Velocity and Thermal Conductivity for CF Flakes

We investigated the sound velocity and anisotropic thermal conductivity of CF flakes using an ultrafast optical pump‐probe technique known as time‐domain thermoreflectance (TDTR) [75]. To measure through‐plane thermal conductivity, we employed a frequency‐modulated pump beam and a time‐delayed probe beam in a spatially overlapped geometry, referred to as co‐aligned TDTR. For in‐plane thermal conductivity measurements, we utilized a beam‐offset technique [76], which involves spatial separation between the pump and probe beams at a fixed time delay. The sound velocity of the longitudinal acoustic phonons propagating along the through‐plane direction was measured by picosecond acoustics [77]. Experimental details are provided in the Experimental Section.

#### 2.2.1 Sound Velocity of CF Flake

**FIGURE 2:**
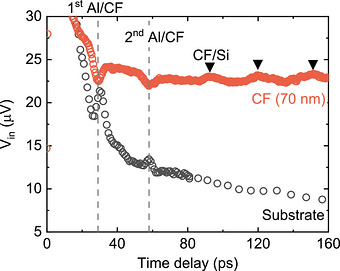
Picosecond acoustics data of a 70 nm‐thick CF flake (red) and of its substrate (gray). The *y*‐axis is the in‐phase TDTR signal (V_in_). The gray dashed lines are the first and second acoustic echoes reflected at the Al/CF interface, and the black triangles indicate the acoustic echoes reflected at the CF/Si interface. The data of the Si substrate is offset by −55 µV.

We determined the longitudinal sound velocity (*v*_L_) of a 70 nm‐thick CF flake using the measured time delay of acoustic echo (Figure 2A, red symbol) and the thickness measured by AFM. The longitudinal sound velocity of CF for the through‐plane direction is (2.22 ± 0.16) nm ps^−1^, approximately 50% lower than that of graphite [78]. The longitudinal elastic constant for the through‐plane direction (*C*_33_) was derived to be *C*_33_ = *ρv*_L_^2^ = (12.3 ± 1.9) GPa when using the bulk CF density of 2.5 g cm^−3^, which is three times lower than that of graphite, i.e., *C*_33_ = (36.5 ± 0.1) GPa [79]. The estimated *v* and *C*_33_ are also lower than those of other 2D materials, such as MoS_2_ (*v*_L_ = 3.2 nm ps^−1^, *C*_33_ = 52 GPa [11]). Instead, these values are rather comparable to those of polymer materials with thermal conductivity of < 0.5 W m^−1^ K^−1^ [80]. Notably, the longitudinal sound velocity of CF is slightly lower than that of PCBM, i.e., (2.3 ± 0.1) nm ps^−1^, a polymer with one of the lowest known thermal conductivities, i.e., (0.030 ± 0.003) W m^−1^ K^−1^ [81]. We did not observe any clear acoustic signals in thicker CF flakes, partly due to the smaller amplitude of acoustic echoes.

#### 2.2.2 Through‐Plane and In‐Plane Thermal Conductivity of CF

**FIGURE 3:**
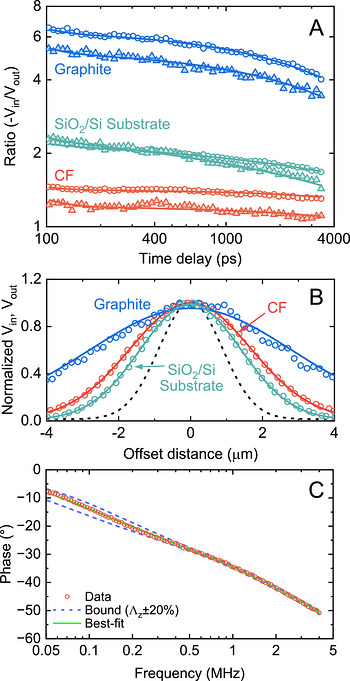
Thermal conductivity measurements of a 178 nm‐thick CF flake (red) compared to those of a graphite flake (blue) and a SiO_2_(500 nm)/Si substrate (green), all of which have been coated with the same Pt film. (A) Through‐plane TDTR data at the modulation frequency (*f*_mod_) of 1.8 (open circles) and 10.9 MHz (solid triangles) and spot size (*w*_0_) of 1.2 µm. The solid lines are the best fit. (B) In‐plane TDTR data (V_out_) at *f* = 1.8 MHz and time delay of −100 ps (open circles). The solid lines are the Gaussian fit of the TDTR data, and the dashed lines are the measured V_in_, indicating the intensity profile of the pump and probe. (C) FDTR data (open circles) at the frequency range of 0.05–4 MHz fitted with the best‐fit of thermal model (green solid line) and Λ_z,CF_ ± 20% bounds (blue dashed lines).

For through‐plane thermal conductivity measurements, we employed the co‐aligned TDTR on CF flakes with the thicknesses of 53–243 nm and the smooth surfaces, i.e., root mean square surface roughness < 15 nm, confirmed by AFM (see Figure S1). We obtained the ratio data of in‐phase to out‐of‐phase voltage (−V_in_/V_out_) signals as a function of time delay and compared the data with a thermal model. Figure 3A shows the measured ratio signals (symbols) and the best‐fit of the thermal model (solid lines) for the 178 nm‐thick CF flake at the modulation frequencies (*f*_mod_) of 1.8 (open circles) and 10.9 MHz (solid triangles), and spot size of the correlated pump and probe beam (1/e^2^ radius) of *w*_0_ ≈ 1.2 µm. The same TDTR measurement was performed on a 20 nm‐thick graphite flake as well as the 500 nm‐thick SiO_2_/Si substrate on which both the CF and graphite flakes were placed. All the samples were coated with a Pt transducer.

The thermal model requires material parameters, including the thermal conductivity (Λ), volumetric heat capacity (*C*), and thickness (*t*) for each layer. To reduce the uncertainty of the free parameters, i.e., through‐ and in‐plane thermal conductivity (Λ_z_ and Λ_r_) of CF, it is crucial to use the other material parameters with high accuracy in the model. For CF, we measured the thickness using AFM and used a heat capacity value of 2.01 J cm^−3^ K^−1^ from the literature [51]. We note that the *t*_CF_ and *C*_CF_ have negligible sensitivity in thermal analysis and thus do not significantly impact the results of Λ_CF_. In case of *C*_CF_, we obtained the identical results for Λ_CF_, even applying the *C*_graphite_, i.e., 1.61 J cm^−3^ K^−1^. Detailed discussion for the sensitivity uncertainty analyses for TDTR experiments is available in Note S1 and Figures S3 and S4.

To measure in‐plane thermal conductivity, we employed the beam‐offset TDTR on the equivalent CF flakes. The beam‐offset TDTR measures the accumulated lateral heat spreading as a function of the offset distance between the pump and the probe beams. We measured the V_out_ signal at negative *t*_d_ (‐100 ps) and low *f*_mod_ (1.8 MHz), where the in‐plane heat diffusion is pronounced. We analyzed the full‐width‐at‐half‐maximum (FWHM) of V_out_ for the given 1/e^2^ spot size, which was measured by V_in_ at positive *t*_d_ = +100 ps and high *f*_mod_ = 10.9 MHz [76, 82]. To extract the in‐plane thermal conductivity, we fitted the measured FWHM to the prediction from a thermal model based on the material parameters and the measured spot size. Figure 3B presents the lateral heat spreading data for CF, graphite, and the SiO_2_/Si substrate, along with the Gaussian fits to the data as solid lines. The black dashed line indicates the cross‐correlated beam size of the pump and probe.

By using co‐aligned and beam‐offset TDTR, we determined the through‐ and in‐plane thermal conductivity of CF with various thicknesses. The analyses of both co‐aligned and beam‐offset TDTR treated the through‐ and in‐plane thermal conductivity of CF as the two free parameters. We fixed the *G* of the top interface, i.e., Al/CF or Pt/CF, as 10 MW m^−2^ K^−1^ based on the best‐fit from the simultaneous analysis of co‐aligned TDTR and FDTR data. In both measurements, the sensitivity to this value is negligible, which has virtually no impact on the results. The *G* of the bottom interface was fixed as 50 MW m^−2^ K^−1^, which is previously reported in literature [83], but the sensitivity to this value is also negligible due to the shorter *d*_p_ (≈ 20–50 nm) compared to CF thicknesses. We note that a similar calculation curve under *f* = 10.9 MHz can be achieved by assuming an ultralow thermal conductance of the top interface, i.e., *G* = 1 MW m^−2^ K^−1^ (see Figure S5). By employing the heat resistance model, a total heat resistance combining G = 1 MW m^−2^ K^−1^ with Λ_z_ = 6 W m^−1^ K^−1^ of the flake produces the equivalent thermal resistance to a flake with ultralow through‐plane thermal conductivity of 0.02 W m^−1^ K^−1^. However, we confirmed that the measured properties are contributed by the entire layer of CF, not its interface, through TDTR with *f* = 1.8 MHz and FDTR, in which the sensitivity to *G* of the top interface is significantly reduced. Furthermore, we independently investigated the effect of fluorination on the interface. A detailed discussion of interfacial contribution is presented in Section 2.3.

To further validate the thermal conductivity of the CF samples, FDTR measurements [84, 85] were performed on the 178 nm thick flake (see Experimental Section (Methods)). Since the sensitivities of the FDTR phase signal to Λ_z_ of the CF are considerably higher than TDTR, especially when *f*_mod_ is in 0.05–0.5 MHz, the uncertainty from FDTR is expected to be smaller (Figure S6). Figure 3C displays the experimental phase data (symbols) alongside the best‐fit curves from the transfer matrix thermal model, where only Λ_z,_ and Λ_r_ of the CF flake were treated as adjustable parameters (solid lines) of a representative measurement. All other model parameters were fixed to the same values used in the TDTR analysis to ensure consistency. We found Λ_z, CF_ = (0.029 ± 0.010) and Λ_r, CF_ = (4.2 ± 1.6) W m^−1^ K^−1^, respectively, which are in good agreement with those determined by TDTR.

**FIGURE 4:**
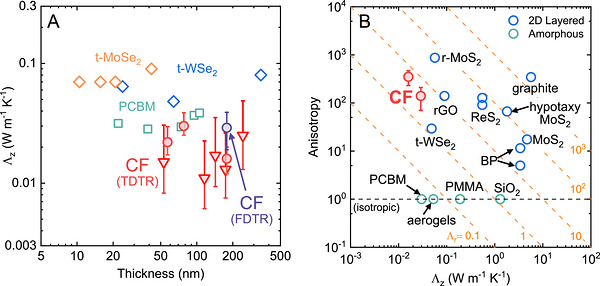
Thermal conductivity of CF compared with other materials. (A) Extremely low through‐plane thermal conductivity (Λ_z_) of graphite fluoride (CF) compared to turbostratic WSe_2_ (Blue diamonds) [7] and MoSe_2_ (orange diamonds) [86], and PCBM (green squares) [81]. Red circles and triangles represent the TDTR measurement with the Pt and Al transducer, respectively. The purple circle represents the FDTR measurement with the Pt transducer. (B) Anisotropy ratio of thermal conductivity of CF (red open circles) as a function of Λ_z_, plotted with other 2D layered materials (blue circles) [7, 38, 86, 87, 88, 89, 90, 91] and amorphous materials (green circles) [81, 92, 93]. Orange dashed lines indicate the constant in‐plane thermal conductivity (Λ_r_).

Figure 4A presents the through‐plane thermal conductivity (Λ_z_) of CF flakes with the thicknesses of 53–243 nm, compared with other solids having ultralow thermal conductivity, including turbostratic WSe_2_ [7] and MoSe_2_ [86] (t‐WSe_2_ and t‐MoSe_2_), and PCBM [81]. The CF flakes exhibit unexpectedly low Λ_z_ values ranging from $0.011_{-0.006}^{+0.011}$ to $0.030_{-0.05}^{+0.09}$ W m^−1^ K^−1^, representing the lowest thermal conductivity reported for any fully dense solid. Using the beam‐offset technique, we derived the in‐plane thermal conductivity ranging from (4.2 ± 1.6) to (5.6 ± 1.4) W m^−1^ K^−1^ for a 178 nm‐thick CF flake. Figure 4B compares the measured through‐plane (Λ_z_) and in‐plane (Λ_r_) thermal conductivities of CF flakes with those of other 2D layered materials (blue open symbols) [7, 38, 86, 87, 88, 89, 90, 91] and amorphous materials (green open symbols) [81, 92, 93] at room temperature. The red shaded region represents the range of thermal conductivity values measured for CF flakes, including associated uncertainties. Among 2D layered materials, CF exhibits a relatively high anisotropy ratio of thermal conductivity exceeding 100, primarily due to its exceptionally low through‐plane thermal conductivity (Λ_z_).

The unexpectedly large reduction in the Λ_z_ and Λ_r_ of CF compared to graphite suggests that CF flakes may have significant structural disorders, necessitating atomic‐level structural investigations. The Λ_r_ of CF is up to 350 times lower than that of the exfoliated graphite flake (1500 ± 200) W m^−1^ K^−1^, as we measured in this work. This marked reduction in Λ_r_ of CF is much more exaggerated than the theoretical prediction of Λ_r_ reduction in the CF monolayer, i.e., Λ_r_ = 145 W m^−1^ K^−1^ [33], only six times smaller than that of graphite. Meanwhile, the Λ_z_ of CF shows an even more dramatic reduction, up to 500 times lower than that of graphite, i.e., (5.3 ± 0.3) W m^−1^ K^−1^. This reduction in Λ_z_ can be partially attributed to weak interlayer vdW interactions caused by fluorine incorporation [52], resulting in reduced *v*_L_ in the through‐plane direction. However, this factor alone cannot fully explain the exceptionally low thermal conductivity observed in CF. Also, the Λ_z_ of CF exhibits over 10‐fold reduction from the minimum thermal conductivity value of 0.36 W m^−1^ K^−1^ at room temperature [94], calculated using the measured sound velocity of CF and graphite's Poisson's ratio, i.e., 0.19 [95]. Rather, the Λ_z_ of CF is comparable to that of polycrystalline MoS_2_ films with interlayer rotations (r‐MoS_2_), i.e., Λ_z_ = 0.05 W m^−1^ K^−1^, though slightly lower, which are known to severely restrict through‐plane thermal transport [88]. This suggests that the thermal transport properties of CF deviate even further from those expected in materials with normal stacking arrangements. Thus, we performed the structural analysis of the exfoliated CF flakes, which we discuss in detail in Sections 2.4 and 3.

### 2.3 Interfacial Thermal Conductance (G) of Surface‐Fluorinated Graphite Flakes

For understanding the origin of extreme heat insulation observed in the CF samples coated with a metal transducer, we sought to differentiate between the contributions of the metal/CF interface and of the bulk CF layer. Since the distinction of the thermal insulation effects of the interface and the bulk CF layer is ambiguous in the measurement of the CF flakes, we separately prepared the pristine graphite flakes for which only the top surface layer was selectively fluorinated. The evaluation of the interfacial thermal conductance of these graphite samples allows us to determine the influence of fluorination on the interface.

**FIGURE 5:**
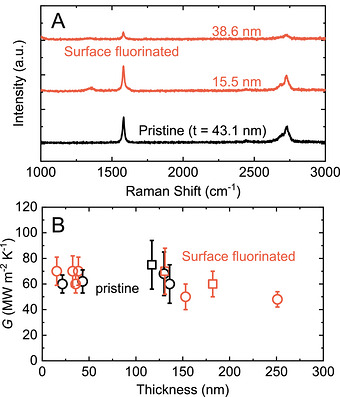
Interfacial thermal conductance (*G*) measurements of a surface fluorinated graphite compared to a pristine graphite flake coated with the Pt and Al film. (A) Raman spectra of a pristine graphite and surface fluorinated flakes with different thicknesses. (B) *G* of surface fluorinated (orange symbols) or pristine graphite (black symbols) and metal transducer, i.e., Pt and Al. Open circle and square symbols indicate the *G* with Al and Pt transducer, respectively.

The surface‐fluorinated graphite flakes were prepared by conducting an XeF_2_ etching process [96, 97] on graphite, which is known to fluorinate only the top surface but not the graphite layers below. The surface coverage of the fluorination is adjusted by the number of etching cycles or fluorination time. Pristine graphite flakes with various thicknesses of 16–182 nm were exfoliated onto SiO_2_ (285 nm‐thick)/Si (1 0 0) and Si (1 0 0) with native oxide substrates and exposed to XeF_2_ gas with a varying number of cycles from 5 to 20 cycles. As shown in Figure 5A, Raman spectra confirmed the surface fluorination of the graphite flakes by the weak presence of a *D* peak near 1350 cm^−1^. We note that Raman spectroscopy has a penetration depth of about 50 nm [98], equivalent to about 150 layers; the Raman signals from the fluorinated surfaces were not dominant in the measured spectra. The effect of XeF_2_ etching was more pronounced in the Raman spectra of monolayer graphene, as shown in Figure S7. The progressive XeF_2_ processing cycles induced gradual structural changes through fluorine attachment to the top surface, as evidenced by the attenuation of the *2D* band (∼2700 cm^−1^) and emergence of the *D* band (∼1350 cm^−1^). As surface fluorination was saturated beyond 10 etching cycles, we selected these flakes for further analysis.

We performed co‐aligned TDTR on the surface‐fluorinated graphite flakes after coating them with a 70 nm‐thick Pt or 80 nm‐thick Al film. We set the through‐plane thermal conductivity (Λ_z_) of graphite and thermal conductance (*G*) of the metal/graphite interface as the free parameters in the thermal model. The in‐plane thermal conductivity (Λ_r_) of graphite was determined from our previous measurements of beam‐offset TDTR, and the heat capacity was taken from literature [99]. The thicknesses of graphite flakes were measured by AFM. By fitting a thermal model to the measured ratio data (Figure S8), Λ_z_ of graphite exhibits thickness‐dependent thermal conductivity, i.e., 1.7–3.2 W m^−1^ K^−1^ for the thickness of 21–43 nm and 5.3–6.7 W m^−1^ K^−1^ for the thickness of 117–250 nm, in accordance with the previous calculations [100, 101]. Meanwhile, *G* remained in a similar range for both pristine and surface‐fluorinated graphite samples (Figure 5B), from 40 to 80 MW m^−2^ K^−1^ with no clear dependency on thickness. The *G* values in this work align with typical *G* values of Al/highly oriented pyrolytic graphite (HOPG), ranging from 40 to 80 MW m^−2^ K^−1^ [102, 103]. Prior work reported that fluorination on monolayer graphene also exhibits no impact on *G* of Au/graphene, while oxidation and nitrogenation increase *G* by up to 40% [104]. Although strong C─F bonds would enhance through‐plane thermal transport, fluorination decreases surface energy, weakening adhesion of Au/graphene and thus leaving *G* unchanged. In contrast, oxidation and nitrogenation increase surface energy, inducing higher thermal conductance. Aligned with prior findings, our work suggests that surface fluorination has minimal impact on thermal transport at the interface, and the CF layer is responsible for the apparent ultralow thermal conductivity. Meanwhile, we expect the *G* of Pt/CF interface to be lower than the *G* between metal and surface‐fluorinated graphite. This is because the former case has double‐sided fluorination as well as strong surface irregularity, as can be seen from the TEM analysis, while the latter undergoes fluorination on one single‐sided surface of graphite. The modified atomic arrangement and presence of the disorders in CF are likely to change the phonon density of states and thus transmission in the CF near the interface.

### 2.4 TEM Analysis on Mechanically Exfoliated CF Flakes

**FIGURE 6:**
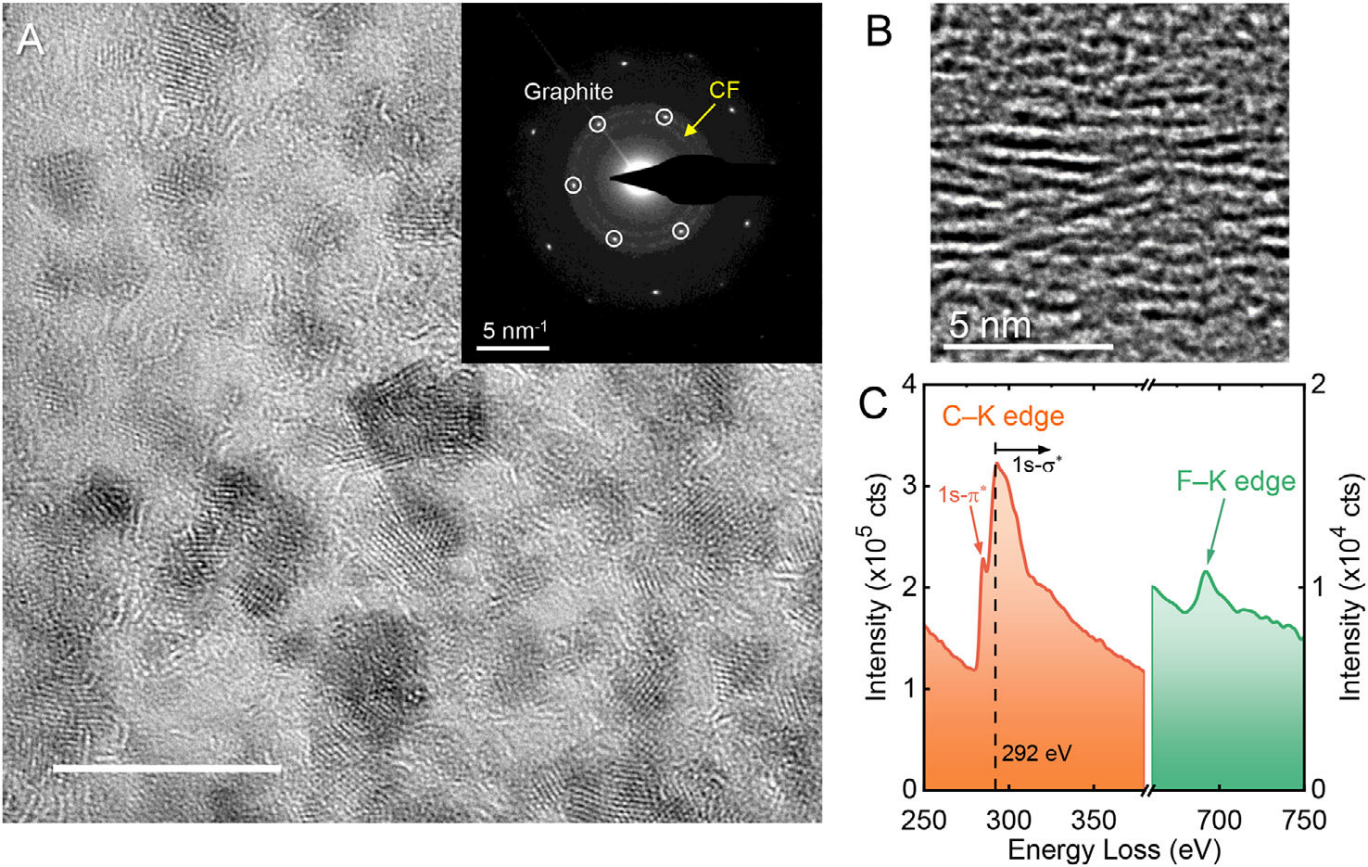
Atomic structure of exfoliated CF flake. (A) High‐resolution TEM image of a CF flake. Inset: selected area electron diffraction (SAED) patterns of the CF flake. (B) Cross‐sectional TEM image. (C) EELS spectra obtained from cross‐sectional TEM. The left side of the graph (red) indicates the C–K edge, and the other (green) indicates the F–K edge.

For analysis of the atomic structures of exfoliated CF flakes, TEM and corresponding selected area electron diffraction (SAED) pattern images were obtained as shown in Figure 6A (See Experimental Section for experimental details). The fluorinated graphite grains with random crystallographic orientations are randomly distributed throughout the single‐crystalline graphite lattice at intervals within approximately 5 nm, demonstrating the highly disordered atomic structure of CF. A similar structure was also observed in scanning transmission electron microscopy (STEM) in Figure S9. As illustrated in the SAED pattern (the inset of Figure 6A), the CF occupies the inner ring patterns (yellow arrow) rather than the hexagonal spots of graphite (white circles), confirming that CF has increased interatomic distances. The in‐plane lattice constants derived from these patterns measure 2.49 Å for graphite and 2.58 Å for CF, consistent with literature values [50, 52]. The distinction between single‐crystal graphite with well‐aligned crystallinity and CF with random crystal orientation is evident in their interatomic distances and regularity of crystal structures. The ring pattern with an interatomic distance identical to the hexagonal spots of graphite is likely attributed to additional grains of graphite with different orientations formed during the fluorination process or to the reduction of fluorine atoms induced during the preparation and observation of the TEM sample. However, these occurrences are rare, as evidenced by their blurred diffraction patterns compared to those of single‐crystalline graphite. The increased interlayer spacing in CF due to F atoms alters the *z*‐axis focus of TEM, resulting in contrast differences between CF and graphite. When focusing on CF to examine its crystal structure, the graphite lattice may appear unclear due to being out of focus, not due to amorphousness. The contrast of CF varies due to differences in the thickness of each grain, while the surrounding graphite exhibits uniform bright contrast in Figure 6A, aligning with the cross‐section TEM image in Figure 6B. CF exhibits a layered structure similar to graphite but contains numerous grain boundaries where nanograins are not stitched, and it features variable interlayer distances ranging from 4.8 Å to 5.8 Å, as illustrated in Figure 6B. To verify that the fluorine atoms were not lost during sample transfer or the focused ion beam process, and that the chemical bonds within CF remained intact, EELS spectra were collected from the corresponding region in Figure 6B, see Figure 6C. The destruction of the 1s‐π^*^ peak in the C–K edge EELS spectra of CF, compared to graphite [105], indicates the fluorination‐induced transformation of carbon hybridization from sp^2^ to sp^3^ [105, 106], corroborating our XPS findings.

## 3 Discussion

The record‐low Λ_z_ of CF flakes, i.e., < 0.030 W m^−1^ K^−1^, even approaches that of air, i.e., 0.02 W m^−1^ K^−1^, primarily due to their highly disordered and inhomogeneous structures formed by fluorination. Although the introduction of fluorine weakens the interlayer vdW forces, this mechanism alone cannot explain the 200–400‐fold decrease in Λ_z_ of CF compared to graphite, as previous calculations demonstrated interlayer binding energy was reduced only approximately twice by fluorination [52]. Rather, for understanding the effect of functionalization, we can refer to the cases of GO and rGO, suggesting that thermal conductivities are dominated by defects or disorders. The thermal conductivity of rGO films is twofold lower in the through‐plane direction (Λ_z_ = 0.09 W m^−1^ K^−1^) compared to GO films, primarily due to pores generated between layers by oxygen and CO_2_ gas during the reduction process of GO films [38]. However, unlike rGO, CF flakes exhibit densely packed structures without visible pores, as shown in TEM images. Instead, CF flakes display two distinctive disorder types affecting thermal conductivity: (1) mass and rotational disorders from randomly oriented nanometer‐sized fluorinated grains, and (2) large variations in interlayer spacing, inevitably accompanied by (3) atomic configurational and stacking disorders. These features resemble those of previously studied layered materials with ultralow thermal conductivity, yet our CF demonstrates even lower values.

To properly contextualize the exceptional properties of CF, we compare CF to layered materials with ultralow through‐plane thermal conductivity of approximately 0.05 W m^−1^ K^−1^ and elucidate why CF occupies even lower thermal conductivity. Designing such materials has been extensively investigated through experiments and calculations, beginning with turbostratic WSe_2_ (0.048 W m^−1^ K^−1^) [7]. Research has demonstrated that extremely low thermal conductivity can result from rotational (or stacking) disorder, as evidenced by randomly stacked MoS_2_ (r‐MoS_2_) experimentally exhibiting around 0.05 W m^−1^ K^−1^ while maintaining decent in‐plane thermal conductivity [88]. Atomistic modeling of graphitic systems also suggests that randomly stacked graphite can achieve the thermal conductivity below 0.1 W m^−1^ K^−1^ in the through‐plane direction [107]. Several studies have identified the reduction in group velocity and lifetime of transverse acoustic (TA) phonon modes as key factors for such ultralow thermal conductivity [86, 108, 109, 110].

Upon fluorination, CF also features randomly rotated interfaces, with the additional effect of fluorine incorporation. The introduction of the heavier atoms reduces the group velocity of heat‐carrying LA and TA phonon branches, resulting in decreased through‐plane thermal transport [111]. We observed a notable 50% reduction in the LA group velocity (*v*_L_) of CF, partially attributed to fluorine incorporation. This significant reduction is separated from the effects of interlayer rotation, which is known to significantly reduce the group velocity of TA branches but only weakly affect that of LA branches, i.e., less than 10% reduction in *v*_L_ of both graphite and MoS_2_ [108, 109]. Moreover, CF differs fundamentally from previously studied ultralow thermally conductive layered materials that maintain well‐defined layered structures. In contrast, CF possesses poorly defined layers, evidenced by large deviations in interlayer spacing, which may further reduce the group velocity of LA phonons, inducing low‐end through‐plane thermal conductivity.

Fluorination at high temperatures can energetically induce various atomic configurations and stacking sequences, which is the intrinsic origin of extreme heat insulation of CF, also partly resulting in the large deviations of interlayer spacing and random orientations. As discussed in Section 1, CF can form four fundamental atomic configurations, with a formation energy of 0.1 eV per atom [23]. Moreover, the formation energy differences of AA, AB, ABC, and AA’ stacking of chair configurations exhibit up to 0.02 eV per atom [52]. Typically, CF dominated with sp^3^ bonding is synthesized at around 600°C [112], i.e., ≈ 0.08 eV, where the thermal energy enables transitions among different fluorine configurations and stacking sequences. Meanwhile, the observed broad interlayer spacing distribution likely originates from disorder in the stacking sequence. This is because the interlayer spacing can increase from 5.70 to 5.92 Å when the stacking sequence changes from AB to AA', with a corresponding formation energy difference of only 0.01 eV per atom [52]. These structural disorders, from atomic disorders to defective microstructure, the extremely low thermal conductivity of CF and also suggest the need for applying glass transport theories [113] to CF, beyond the traditional minimum thermal conductivity model for amorphous materials [94]. In summary, this highly disordered nanostructure suggests that CF exhibits different underlying origins compared to other ultralow thermally conductive layered materials, enabling it to achieve record‐low thermal conductivity. To further elucidate the thermal transport mechanisms governing the ultralow thermal conductivity of CF, temperature‐dependent TDTR measurements are an essential next step. Such measurements would help distinguish between disorder‐dominated scattering, which is expected to yield a weak or negligible temperature dependence, and anharmonic phonon‐phonon scattering, which would produce a characteristic 1/T behavior.

The significant reduction Λ_r_ of CF, i.e., 4.2–5.6 W m^−1^ K^−1^, is likely due to the inhomogeneously dispersed CF grains. This value is notably lower than both theoretical predictions, i.e., 145 W m^−1^ K^−1^ [33], and experimental measurements of monolayer CF, i.e., about 80 W m^−1^ K^−1^ [57]. As discussed above, inhomogeneous fluorination results in broken layers within the graphite flake, forming the non‐uniform boundaries or edges between the CF and C. These boundaries or edges dominate the suppression of the thermal transport in the in‐plane direction. Furthermore, the fluorinated grains contain the disorder of fluorine configurations as discussed above, which further reduces the in‐plane thermal conductivity. For comparison, GO film exhibited a lower Λ_r_, i.e., 2.9 W m^−1^ K^−1^, which is mainly attributed to the lateral dimensions of ordered clusters in microstructure [38]. However, the average length of the ordered clusters in the GO film was estimated to be 3.5 nm, which is similar to that of the CF flakes. Despite similar grain sizes between CF and GO, CF exhibits higher Λ_r_ values than GO film. This difference originates from their stoichiometric compositions; CF possesses a near‐stoichiometric composition, whereas GO does not. GO contains various functional groups, including hydroxyl, carbonyl, and carboxyl groups, which irregularly attach to the carbon backbone and disrupt the honeycomb carbon layer [21]. This disruption weakens the in‐plane C─C bonds and leads to a significant reduction in the Λ_r_ of GO.

The dramatic reduction in thermal conductivity of CF presents promising opportunities for thermal insulation applications, in contrast to its previous prominence as a thermal management material. As discussed in the Introduction, CFs have been employed in previous studies as high thermal conductivity fillers for thermal interface materials [46, 47, 48, 49]. A recent study demonstrated the application of monolayer CF as a heat sink for graphene field‐effect transistors by forming lateral heterostructures, which reduced the peak hotspot temperature by approximately 120 K compared to configurations without a heat sink [114]. However, CF in this work exhibits record‐low through‐plane thermal conductivity and substantially reduced in‐plane thermal conductivity, making it suitable for thermal insulation from external environments [115] or preventing thermal runaway in battery devices [116, 117]. Remarkably, CF is a fully dense solid material with the elastic constants (C_33_) of (12.3 ± 1.9) GPa. This indicates superior mechanical properties compared to conventional thermal insulation materials such as aerogels, which exhibit elastic moduli that are one to two orders of magnitude lower [118].

## 4 Conclusion

In this study, we characterized the structural and thermal properties of mechanically exfoliated CF flakes and reported the extreme thermal conductivity along the through‐plane direction and thus an extremely high anisotropy ratio. The through‐plane thermal conductivity was measured as < 0.030 W m^−1^ K^−1^ for the CF flakes with the thicknesses of 53–243 nm, the lowest value that has been reported for fully dense solids. The extremely limited thermal transport for the through‐plane direction stems from the densely packed fluorinated grains with rotational disorders and a strong reduction of the longitudinal sound velocity for the through‐plane direction. The interlayer mass mismatch between the fluorinated grains and pristine graphite within CF flakes is further expected to limit thermal transport across the rotated interface with the adjacent graphite layer. Besides, the large deviation of interlayer spacing of CF, which appeared from configurational and stacking disorders, is a distinctive feature that limits thermal transport in the through‐plane direction. The in‐plane thermal conductivity was 4.2–5.6 W m^−1^ K^−1^, resulting in an anisotropy ratio exceeding 100. The reduction in the in‐plane thermal conductivity is mainly due to the inhomogeneously formed boundaries between the CF and C. The record‐low thermal conductivity and its correlation with structural properties in CF offer valuable insights into the understanding of thermal transport properties of graphite derivatives. The distinctive combination of compositional and structural properties is further expected to open new avenues as thermal insulation materials in various applications.

## 5 Experimental Section (Methods)

### 5.1 Preparation for CF Samples

Graphite fluoride (CF) flakes were mechanically exfoliated from commercially available powder (ACS Materials, GT1FF012) onto substrates of wet oxidized SiO_2_(500 nm)/Si and native SiO_x_/Si. Prior to exfoliation, the substrates were cleaned with acetone and isopropyl alcohol (IPA) under ultrasonication for 5 min.

### 5.2 Preparation for XeF_2_‐Treated Graphite Samples

Graphite flakes were mechanically exfoliated from (NGS Trading & Consulting GmbH, Germany) onto the substrate of wet oxidized SiO_2_(285 nm)/Si and native SiO_x_/Si. For the fluorination of graphite, the home‐built XeF_2_ etching system (SCEN(Scientech), Korea) was used. With a pulsed flow mode of the XeF_2_ etching system, the degree of fluorination of the graphite was controlled by adjusting the number of processing cycles in the system. The pressure and processing time for each cycle were fixed at 50 mTorr and 1 s.

### 5.3 Raman Spectroscopy

Raman spectroscopy (JASCO NRS‐4500) was performed under ambient conditions to assess the degree of fluorination of graphite. A 532 nm laser with an approximate spot size of ≈ 1 µm served as the excitation source. To minimize any potential degradation of the sample from laser exposure, the laser power was kept below 5 mW, with an acquisition time of 30 s.

### 5.4 Field Emission Scanning Electron Microscopy–Energy X‐Ray Spectroscopy (FESEM‐EDS)

FESEM‐EDS (JSM‐7600F) was operated at an accelerating voltage of 15 kV, with a working distance of 8 mm. EDS analysis was conducted at the selected area to determine the elemental composition, with a minimum acquisition count of over 100 000 in a spectrum.

### 5.5 X‐Ray Photoelectron Spectroscopy (XPS)

XPS (VersaProbe III) analysis was performed to measure the composition and bonding configuration of the surface of graphite fluoride flakes. The XPS spectra were acquired via an Al Kα source (1486.6eV) operating at 72W. The C1s and F1s peaks were deconvoluted by employing a Gaussian–Lorentzian sum function via CasaXPS software.

### 5.6 TEM Sample Preparation and Characterization

CF Flake was transferred to a holey carbon Au TEM grid using a poly (methyl methacrylate) (PMMA)–based wet‐transfer method. PMMA was spin‐coated on graphite fluoride/Si substrate, and the edges of the substrate were scoured before immersion in a 5 wt.% KOH solution. The PMMA/graphite fluoride film detached and floated on the surface of the solution. The film was rinsed with deionized water three times, each for 30 min, before being transferred onto a holey carbon Au TEM grid. The PMMA was removed by soaking the TEM grid in acetone for 6 h. TEM, STEM, and SAED pattern images were acquired using Cs‐corrected monochromated TEM instruments (Thermo Fisher, Themis Z, and JEOL, JEM‐ARM 200F) operating at 80 kV. For cross‐sectional TEM, the samples were prepared by a focused ion beam (Thermo Fisher, Helios G4), with the protective layer of Pt.

### 5.7 Time‐Domain Thermoreflectance (TDTR)

For the evaluation of thermal transport properties of CF and XeF_2_‐treated graphite flakes, we used TDTR, a pump‐probe optical technique that uses an ultrafast laser as the light source. A mode‐locked Ti: Sapphire laser (Mai Tai HP) with a wavelength centered at 781 nm and a repetition rate of 80 MHz, and modulate the pump beam by an electro‐optic modulator (EOM) at the frequency (*f*). By varying *f* and spot size (1/e^2^ radius, *w*_0_) of the laser beams, we achieved an optimized sensitivity for through‐ and in‐plane thermal conductivity in co‐aligned TDTR and beam‐offset techniques, respectively [119]. We conducted the reproduction of co‐aligned TDTR measurements for experimental validation, as shown in Note S2 and Figure S10. We used *f* of 10.9 MHz for the through‐plane measurement and 1.8 MHz for the in‐plane measurement. We used *w*_0_ of 1.2–1.3 µm in TDTR experiments, which was independently determined in each sample at the high modulation frequency of *f* = 10.9 MHz and time delay of *t*_d_ = +100 ps, where the TDTR signal dominantly depends on the *w*_0_. Detailed information on spot size determination is available in Note S3 and Figures S11 and S12. We also confirmed that the heat loss into the ambient environment is negligible, see Note S4 and Figure S13.

### 5.8 Picosecond Acoustics

The longitudinal sound velocity along the *z*‐axis within materials can be measured by co‐aligned TDTR, called picosecond acoustics [120]. Pulsed heating of the metal transducer by the pump beam induces a strain pulse in the through‐plane direction. This pulse partially reflects at the interface and returns to the surface and alters the measured thermoreflectance signal, i.e., acoustic echo. The timing of the echo allows the estimation of either the thickness or the sound velocity of the layer, depending on which property is known. The shape of the acoustic echo mostly depends on the ratio of acoustic impedances (*Z*) between the adjacent layers [121]. The acoustic impedance is given by *Z* = *ρv*, where *ρ* and *v* are the mass density and sound velocity, respectively.

### 5.9 Frequency‐Domain Thermoreflectance (FDTR)

The thermal conductivity of the CF sample, previously measured by TDTR, was further validated using FDTR. In the FDTR measurements, a 445 nm pump laser, modulated at frequencies ranging from 0.2 to 4 MHz, was used to periodically heat the CF flakes coated with ≈ 70 nm Pt transducer layer. The resulting temperature oscillations were monitored by a continuous‐wave 532 nm probe laser co‐aligned with the pump spot. Both beams were focused onto the sample through a Mitutoyo M Plan Apo 50 × objective lens to a 1/*e* ^2^ radius of ≈ 1.18 µm, as determined by the knife‐edge method. The reflected probe signal as a function of modulation frequency was recorded using a Thorlabs PDB230A balanced photodetector and demodulated with an HF2LI lock‐in amplifier. The uncertainty analysis of the FDTR measurement results can be found in Note S1, which reports the uncertainties determined by both the Monte Carlo method and statistical analysis based on repeated measurements [119].

### 5.10 Steady‐State Heating in Thermoreflectance Experiments

Due to the low thermal conductivity of CF, it is challenging to control the steady‐state heating temperature rise. TDTR experiments showed the steady‐state heating of the samples up to 17 and 10 K at room temperature for Pt and Al transducers, respectively, while FDTR experiments exhibited a steady‐state temperature rise of up to ≈ 40 K. However, CF is not expected to be strongly temperature‐dependent, and thus all measurements, demonstrating consistency across each other, can be effectively compared at room temperature conditions. We discussed the detailed procedure for estimation of steady‐state temperature rise and validated the sample stability, available in Note S6 and Figure S14.

## Author Contributions

Wonsik Lee and Hyejin Jang conceptualized and designed the project. Wonsik Lee performed structural and thermal characterization (TDTR) of graphite fluoride flakes, with input from Richard B. Wilson. Donghoon Moon and Gwan‐Hyoung Lee carried out sample preparation using XeF_2_ treatment, Raman spectroscopy, and TEM analysis (both top‐view and cross‐sectional). Ziyan Qian and Qiye Zheng performed thermal characterization (FDTR) and its analysis for cross‐validation. Changheon Kim conducted XeF_2_ treatments, while Jinwoo Kim prepared and analyzed top‐view TEM samples. Yeojin Lee performed control measurements. Hyobin Yoo advised on and analyzed the top‐view TEM images. Wonsik Lee and Hyejin Jang analyzed the data and wrote the manuscript.

## Conflicts of Interest

The authors declare no conflicts of interest.

## Supporting information




**Supporting File**: advs75631‐sup‐0001‐SuppMat.docx.

## Data Availability

The data that support the findings of this study are available from the corresponding author upon reasonable request.
