# Supplementary material for: Disorder‐Induced Extremely Low Thermal Conductivity of Graphite Fluoride
Source: Adv Sci (Weinh). 2026 May 8:e18438. Online ahead of print. doi: 10.1002/advs.202518438 (PMC13336073; doi:10.1002/advs.202518438)
Supplement: Supplementary file 1 — Supporting File: advs75631‐sup‐0001‐SuppMat.docx. [file ADVS-9999-e18438-s001.docx]

Disorder Induced Extremely Low Thermal Conductivity of Graphite Fluoride

*Wonsik Lee, Donghoon Moon, Ziyan Qian, Jinwoo Kim, Changheon Kim, Yeojin Lee, Hyobin Yoo, Richard B. Wilson, Qiye Zheng, Gwan-Hyoung Lee, and Hyejin Jang**

W. Lee, D. Moon, J. Kim, C. Kim, Y. Lee, Prof. H. Yoo, Prof. G.-H. Lee, Prof. H. Jang

Department of Materials Science and Engineering

Seoul National University

Seoul, 08826, Republic of Korea

E-mail: [hjang@snu.ac.kr](mailto:hjang@snu.ac.kr)

Prof. R. B. Wilson

Department of Materials Science and Engineering & Mechanical Engineering

University of California Riverside

Riverside, California 92521, USA

Z. Qian, Prof. Q. Zheng

Department of Mechanical and Aerospace Engineering

The Hong Kong University of Science and Technology

Hong Kong, SAR, China

Prof. H. Yoo, Prof. G.-H. Lee, Prof. H. Jang

Research Institute of Advanced Materials

Seoul National University

Seoul, 08826, Republic of Korea

**Supporting Information**

Supporting Information is available from the Wiley Online Library or from the author.

*Characterization of CF Flakes*

**Figure S1.** Atomic force microscopy (AFM) measurements of CF flakes showing **(A,B)** thickness and **(C,D)** thickness distributions. Yellow lines in (A) and (B) indicate the measurement paths for height profiles (shown in insets). Blue line in (B) indicates the range of offset distance in beam-offset TDTR measurements shown in Figure 3B. Pink boxes denote areas used for roughness measurements. In (C) and (D), solid lines represent peak distributions within the flakes, red shaded area are corresponding to TDTR measurement spots. The black dashed lines show the overall thickness distribution envelope.

**Figure S2.** X-ray diffraction (XRD) spectrum (orange line) of an original carbon monofluoride flakes. Open triangles indicate the diffraction peaks of CF, i.e., (001) and (001). Solid triangles indicate the diffraction peaks of graphite, i.e., (002) and (004).

*Supporting Note 1:*

*Sensitivity and Uncertainty Analysis of TDTR and FDTR experiments on CF flake.*

To ensure the reliability of co-aligned TDTR and beam-offset experiments in this work, we conducted a sensitivity analysis of each experiment. The sensitivity of TDTR experiment is defined as

$$\text{S}_{\text{α}}^{\text{signals}}\text{= }\frac{\text{∂(ln(}\text{ }\text{signals}\text{)}\text{)}}{\text{∂(}\ln\left( \text{α} \right)\text{)}}$$

where the “signals” are the ratio (= -V_in_/V_out_) of co-aligned TDTR and FWHM of beam-offset measurements. Here, *α* represents one of the system or material parameters in thermal model. Figure S3A, B shows the sensitivity to ratio of co-aligned TDTR in the conditions of *f* = 10.9 MHz and *w_0_* = 1.2 μm, and Figure S3C presents the sensitivity to FWHM in the conditions of *f* = 1.8 MHz and *w*_0_ = 1.2 μm. For transducer, we used either Al or Pt, and sensitivity analysis of co-aligned TDTR (Figure S3A, B) revealed that the thermal diffusivity, i.e., α = $\sqrt{\text{Λ}_{\text{z}}\text{/C }}$, and thermal mass, i.e., *Ct*, of transducer dominate the TDTR signal. The large influence of the thermal diffusivity and thermal mass of the transducer is attributed to (1) the small *w*_0_ of about 1.2 μm, resulting in non-negligible in-plane heat diffusion within the transducers with thermal diffusion lengths of about 1.5 and 0.7 μm for Al and Pt, respectively, and (2) the short thermal penetration depth (*d*_p_) of CF, i.e., *d_p, z_* = $\sqrt{\text{Λ}_{\text{z}}\text{/}\text{π}\text{C}f_{mod}\text{ }}\approx$20 nm at *f*_mod_ of 10.9 MHz, where Λ_z_, *C*, and *f* are through-plane thermal conductivity, volumetric heat capacity, and modulation frequency, respectively, which stems from the extremely low thermal conductivity ^[1]^. We chose Pt as a transducer instead of a more typical choice of Al to suppress the sensitivity to in-plane heat diffusion within the transducer, achieving higher sensitivity to the Λ_z_ and Λ_r_ of CF in both TDTR experiments. However, the sensitivity to the properties of Pt is still significant compared to the sensitivity to the Λ_z_ of CF in spite of the lower Λ_Pt_ ($\approx$40 W m^-1^ K^-1^) than that of Al ($\approx$180 W m^-1^ K^-1^). We note that although the sensitivity to the Λ_z_ of CF appears negligible, this observation is mainly attributed to the ultralow value of the Λ_z_ of CF. We fixed the interfacial thermal conductance between the metal transducer (Al or Pt) and CF (*G*_1_) as 10 MW m^-2^ K^-1^ in the sensitivity analysis, determined by the cross-validation between the co-aligned TDTR and FDTR measurements. For the thermal conductance of bottom interface at between the CF and substrate (*G*_2_) was fixed as 50 MW m^-2^ K^-1^, based on the literature ^[2]^.

We carefully characterized the material parameters of metal transducers to minimize their significant influence on the thermal model with the sensitivity analysis (Figure S3A,B). We carefully determined the properties of metal transducer Al and Pt: Λ_Pt_ from 4-point-probe measurement of electrical resistivity and the Wiedemann-Franz law, *t*_Al_ or *t*_Pt_ from picosecond acoustics and AFM, and *C*_Al_ or *C*_Pt_ from the literature ^[3-4]^. For Pt, we incorporated a 6% phonon contribution to the Λ_Pt_ ^[5]^, since Wiedemann-Franz law applied to electrical resistivity accounts only for thermal conduction by electrons. The properties of metal transducers were further validated by modeling beam-offset TDTR measurements on 500-nm-thick SiO_2_ on Si substrates coated with the metals.

We have observed weak sensitivity to *C*_CF_, which is due to complementary effect between through-plane effusivity and in-plane diffusivity in 3D heat transport regime. Figure S3C shows the sensitivity of the TDTR ratio signal to Λ_z,CF_, Λ_r,CF_, and *C*_CF_ at modulation frequencies of 10.9 and 1.8 MHz. At both frequencies, the sensitivities to Λ_z,CF_ and Λ_r,CF_ are clearly non-zero, confirming that the measurement is independently sensitive to the through-plane and in-plane thermal conductivities. In contrast, the sensitivity to *C*_CF_ is relatively small due to the cancellation between the through-plane effusivity and in-plane diffusivity contributions under the 3D heat transport regime. This near-cancellation makes the extracted thermal conductivities robust against uncertainty in the heat capacity input.

For 2D layered materials with anisotropy, the anisotropy ratio of thermal conductivity should be considered in the thermal model as the large in-plane thermal conductivity can substantially influence the ratio signal particularly at lower fmod and smaller *w*_0_, e.g., *f*_mod_ of 1.8 MHz and *w*_0_ of 1.2 μm in this work. Figure S3C demonstrates that reducing the *f*_mod_ from 10.9 MHz to 1.8 MHz increases the sensitivity to the in-plane thermal conductivity of CF. However, the sensitivity of the TDTR ratio to the in-plane thermal conductivity is still limited, thus we iteratively modeled the co-aligned and beam-offset TDTR data to simultaneously determine the through- and in-plane thermal conductivities.

We calculated uncertainties of Λ_z_ and Λ_r_ through error propagation in TDTR experiments. The uncertainties were calculated by using an equation as:

$$\text{η}_{\text{Λ}}\text{=}\sqrt{\sum_{\text{α}} {\text{(}\frac{\text{S}_{\text{α}}}{\text{S}_{\text{η}_{\text{Λ}}}}\text{η}_{\text{Λ}}\text{)}}^{2}}$$

where *η* is the uncertainty and α is other modeling parameters except fitting parameter, i.e., Λ_z_ and Λ_r_ of CF in co-aligned TDTR and beam-offset, respectively. We set the errors of Λ, *C*, *t, w_0_*, and phase as ± 3%, ± 3%, ± 3%, +3%, and 0.1° in transducer and substrate materials, respectively. For CF, we set the errors of *C* and *t* as ± 10% and ± 3%, respectively. For the error of CF’s Λ_r_, we set 45 % based on the uncertainty analysis in beam-offset TDTR. We note that the present uncertainty analysis assumes independent parameter perturbations and does not account for correlations between input parameters.

In beam-offset TDTR, we independently considered the uncertainty of FWHM for V_out_ as 0.3%, leading to the uncertainty of 13.9 % in Λ_r_. The uncertainty of FWHM due to other system and material parameters is 42.8% in Λ_r_. Consequently, the total uncertainty of Λ_r_ of CF is $\sqrt{13.9^{2}\text{+}42.8^{\text{2 }}}\text{ \textasciitilde45\%}$.

**Figure S3.** All measurements are conducted at the pump and probe beams with 1/e^2^ radius of about 1.2 μm. Sensitivity of ratio (-V_in_ / V_out_) in co-aligned TDTR at a modulation frequency (*f_mod_*) of 10.9 MHz coated with **(A)** Al on CF(243 nm)/Si and **(B)** Pt on CF(178 nm)/SiO_2_(500 nm)/Si, respectively. **(C)** Frequency dependent behavior of sensitivity of ratio to thermal conductivity of graphite fluoride coated with Pt transducer. Dashed and solid lines are the sensitivity to Λ_z,CF_ (red), Λ_r,CF_ (blue), and *C*_CF_ (green) calculated at *f*_mod_ = 1.8 and 10.9 MHz, respectively. **(D)** Sensitivity of the FWHM of V_out_ in beam-offset TDTR at *f_mod_* = 1.8 MHz and a time delay (*t*_d_) of -100 ps. Λ, *G*, *C*, and *t* represent thermal conductivity, interfacial thermal conductance, volumetric heat capacity, and thickness, respectively.

To comprehensively evaluate the uncertainty of co-aligned and beam-offset TDTR, we performed a Monte Carlo-based joint analysis ^[6]^. The analysis simultaneously analyzes the co-aligned TDTR data at both modulation frequencies (10.9 MHz and 1.8 MHz) and FWHM data from the beam-offset TDTR measurements, and propagates all controlled-parameter uncertainties into the joint confidence region of (Λ_z_, η). The Monte Carlo analysis used 300 iterations, and all uncertainties below are reported at the 1-σ level unless otherwise specified.

As shown in Figure S4A and B, the joint analysis yields the following results: Λ_z_ = 0.016 W m^-1^ K^-1^ with 25% marginal uncertainty. For Λ_r_, we calculated η·Λ_z_ of each iteration, resulting in Λ_r_ = 5.6 W m^-1^ K^-1^ with 25% marginal uncertainty, and a correlation coefficient ρ(Λ_z_, η) = -0.514. Compared to independent single-measurement estimates of 39% and 45% for Λ_z_ and Λ_r_, respectively, the joint analysis tighten out uncertainties by a factor of $\approx$40%. This confirms that the co-aligned and beam-offset TDTR measurements provide complementary constraints that reduce the joint uncertainty, while verifying that neither single-measurement estimate was overstated.

**Figure S4.** Results of Monte Carlo-based joint analysis for uncertainty calculations of **(A)** through- (Λ_z_) and **(B)** in-plane thermal conductivity (Λ_r_) of CF. The histograms are the fitted results from 300 iterations and solid lines represent fitted normal distribution of simulated results.

For FDTR experiments, the sensitivity of FDTR experiments is defined as

$$\text{S}_{\text{α}}^{\text{phase}}\text{= }\frac{\text{∂(}\text{phase}\text{)}}{\text{∂(}\ln\left( \text{α} \right)\text{)}}$$

Where $\text{α}$ represents one of the key material or system parameters in thermal model. Figure S6 shows the sensitivity to phase of FDTR measurement of a 178-nm-thick CF flake at the frequency range of 0.02 to 0.5 MHz and *w*_0_ = 1.18 μm. Both the Λ_z, CF_ and Λ_r, CF_ exhibit considerable sensitivity within the examined frequency range. Notably, within the frequency range of 0.02 to 0.5 MHz, the phase gradient signal is predominantly sensitive to Λ_z, CF_ ​, thereby ensuring the reliability of the FDTR fitting results. Among all parameters, the *C*_Pt_, *t*_Pt_, *w*_probe_, and *w*_pump_ show high sensitivity across the entire frequency range. In contrast, the sensitivities for both the *G*_Pt/CF_, *G_CF/SiO2_* and the Λ_Pt_ were found to be close to zero, indicating a negligible influence on the measurements. The small $S_{\Lambda_{Pt}}^{Phase}$ and large $S_{\Lambda_{z, CF}}^{Phase}$ makes the FDTR experiments relatively more reliable for the study of the CF flake.

For uncertainty calculation in the FDTR, we incorporated three possible uncertainties: (1) standard deviation (*std*) from repeated measurements, (2) statistical uncertainty determined by using the formula expressed as $\frac{std\cdot t_{score}}{\sqrt{n}}$, and (3) error propagation calculated by assuming 3% error for all fixed parameters, which is identical to TDTR uncertainty calculation.

**Figure S5.** TDTR analysis in the perspectives of bulk and interface insulation on 243-nm-thick CF flake shown in Figure 1A, B. **(A)** Sample geometry in each thermal model. The left geometry represents the “bulk fitting” and the other is “interface fitting”, respectively. Λ_z_ and *G*_1_ represent the through-plane thermal conductivity interfacial thermal conductance of Al/CF (black) or Al/graphite (blue), respectively. In the bulk fitting, we fixed the *G*_1_ as 10 MW m^-2^ K^-1^ in the thermal model. **(B)** Measured through-plane TDTR plots. Open symbols are the measured ratio data of the CF flake shown in Figure 1A at the frequency of 10.9 MHz and *w*_0_ = 1.08 μm. Orange and blue solid lines refer to the best-fit of bulk and interface fitting, respectively.

**Figure S6.** Sensitivity of phase in FDTR experiments measured on Pt/CF(178 nm)/SiO_2_/Si at the frequency range of 0.05 to 4 MHz. The measurements are conducted at the averaged spot size between pump and probe beams with 1/e^2^ radius of 1.18 μm.

**Figure S7.** Raman spectra of fluorinated monolayer graphenes with varying processing cycles of XeF_2_-etching. **Figure S8.** Through-plane TDTR data on surface fluorinated graphite flakes with varying thickness, coated with **(A)** Al and **(B)** Pt film. The orange triangles and the black circles in Figure S8A represent the through-plane TDTR data for surface-fluorinated and pristine graphite, respectively. In Figure S8B, the purple and orange triangles represent the data for surface-fluorinated graphite, and the black circles represent the data for pristine graphite. The TDTR measurements conducted at the modulation frequency (*f*_mod_) of 10.9 MHz and spot size (*w*_0_) of 6.0 μm. The solid lines are best fit.

**Figure S9.** Scanning transmission electron micrograph of the exfoliated CF flake shown in Figure 6.

*Supporting Note 2:*

Reproduction of TDTR Measurements and Measurement multiple points at the same flake.

We conducted additional TDTR experiments at multiple points in the CF flake shown in Fig. 1A for reproduction. As shown in Figure S10A, we recognized the maintained ratio signals in five different points which expressed in Figure S10B.

**Figure S10. (A)** Measured ratio signals at five different points and **(B)** bright-field optical micrographs of an Al deposited CF flake exfoliated on a Si substrate, same as Figure 1A. Yellow open circles indicate TDTR measuring spots.

*Supporting Note 3:*

Spot size determination in TDTR experiments.

We determined the spot size *w­*_0_ by conducting beam-offset scanning at the high modulation frequency *f* = 10.9 MHz and time delay of +100 ps, where the TDTR signal dominantly depends on the *w*_0_, as shown in sensitivity analysis (Figure S11). We calculated the uncertainty of spot size based on this sensitivity analysis with assigning identical errors of other materials parameters, giving 0.8% uncertainty in spot size.

The uncertainty of offset-distance in beam-offset TDTR can be another potential source for spot size uncertainty. We calibrated the offset-distance by using microplate and camera calibration, resulting in a 0.1% uncertainty.

The use of a 50× objective lens introduces positioning uncertainty related to sample placement at the focal plane. Specifically, a 1.4 μm deviation from the focal position, corresponding to 0.25 times the Rayleigh length, which results in a 3% increase in spot size. Given that the differential micrometer used for positioning the objective lens has a resolution of 1 μm, this precision reasonably encompasses the possible focusing errors. Therefore, we comprehensively assigned a ±3% uncertainty to the spot size determination.

**Figure S11.** Sensitivity of the FWHM of V_in_ in beam-offset TDTR at *f_mod_* = 10.9 MHz and a time delay (*t*_d_) of +100 ps, which calculated on CF(243 nm)/Si coated with Al.

We used a small *w*_0_ of 1.2–1.3 μm in TDTR experiments, which independently determined in each sample, due to size-limited flat area within the CF flakes. For validation, we conducted TDTR measurement at the rough point in the CF flake, which shown in Figure 1A. The observed data at rough point exhibit anomalous ratio curve profile, as shown in **Figure S12**.

**Figure S12.** Ratio signals of co-aligned TDTR experiments for comparison to the same TDTR measurements at rough area. Orange open symbols and solid line are the data and fitted model at the flat area, while the blue open symbols represent the measured data at rough point. TDTR experimend conducted at *f_mod_* = 10.9 MHz, on CF(243 nm)/Si coated with Al.

*Supporting Note 4:*

Effect of heat loss from the metal transducer into ambient environment

Since Λ_z, CF_ is extremely low and even comparable to that of air ($\approx$0.026 W m^-1^ K^-1^), we investigated the effect of potential heat loss from metal transducer into ambient environment. We modified the boundary condition of the conventional thermal model^[1]^ the top surface of metal transducer by adding the heat loss by convection as:

$$q_{modified}=q_{laser}-h_{conv}\cdot\Theta_{top}$$

Where Θ_top_ is the temperature of the top surface and *h*_conv_ is convection coefficient, and *q*_modified_ and *q*_laser_ indicate the incident heat flux with and without considering convection. We systemically varied *h*_conv_ from 10 to 50 W m^-2^ K^-1^, covering the full range of natural and even forced convection of air^[7]^. We also applied exaggerated values of *h*_conv_, i.e., 1 and 10 MW m^-2^ K^-1^ for validating the heat loss from convection is adequately applied into the modified thermal model.

As shown in Figure S13, ratio curve calculated under reasonable condition of *h*_conv_ = 10 and 50 W m^-2^ K^-1^ (red dashed line) exhibits negligible deviation from the conventional model (blue solid line). Meanwhile, under *h*_conv_ = 1 and 10 MW m^-2^ K^-1^, much higher ratio curves are calculated, ensuring the model reliability.

**Figure S13**. Thermal model considering convection of ambient environment. Blue solid line indicated conventional thermal model. Colored dashed lines indicate the ratio signal calculated from convection-inclusive thermal model with convection coefficient *h*_conv_ = 10, 50 W m^-2^ K^-1^ (red) and 1 and 10 MW m^-2^ K^-1^ (green and purple, respectively).

*Supporting Note 5*:

Validation of sample stability under steady-state temperature rise

The steady-state temperature rise (ΔT_ss_) was estimated by evaluating the thermal model at zero modulation frequency (*f* = 0), using the same multilayer structure and thermal parameters employed in the TDTR fitting^[8]^. Since Pt has approximately 4 times lower thermal conductivity ($\approx$40 W m^-1^ K^-1^) than Al ($\approx$180 W m^-1^ K^-1^), lower laser powers were used for Pt-transducer measurements to minimize heating. For the Pt case (I_pump_ = 1.0 mW, I_probe_ = 0.5 mW, and *w*_0_ = 1.23 μm), the calculated ΔT_ss_ is 17 K; for the Al case (I_pump_ = 2.2 mW, I_probe_= 1.1 mW, and *w*_0_ = 1.08 μm), ΔT_ss_ is 10 K.

To confirm that these temperature rises do not distort the TDTR signals, we conducted power-dependent measurements on the same CF flake. As shown in Figure S14A, V_in_ normalized by pump and probe power remains constant across varying laser powers, confirming that the thermoreflectance response is in the linear regime. The ratio data remains almost unchanged over five repeated measurements on the same spot of CF flake (Figure S14B), confirming sample stability without any signal distortion. Furthermore, the photodiode detector voltage, which is proportional to the transducer reflectivity, exhibits a standard deviation below 0.1% throughout the measurement (Figure S14C), ruling out any transducer degradation or burning. A reference measurement on the adjacent Pt/SiO_2_(500 nm)/Si substrate floor (I_pump_ = 1.4 mW, I_probe_ = 0.9 mW, ΔT_ss_ = 11 K) yielded a fitted SiO_2_ thermal conductivity of (1.40 ± 0.16) W m^-1^ K^-1^, consistent with literature values. This reference measurement, performed on a region with identical Pt reflectivity, further validates that the transducer remains undamaged during CF measurements.

**Figure S14. (A)** V_in_ measurement with varying beam power. Red open circles: I_pump_ = 0.7 mW, I_probe_ = 0.4 mW; black open circles: I_pump_ = 1.0 mW, I_probe_ = 0.5 mW. **(B)** Repeated measurements taken at the same spot on the CF flake. Each open symbol indicates the number of TDTR measurements. **(C)** Trace of measured voltage of photodiode detector during TDTR measurement.

References

[1] P. Jiang, X. Qian, R. Yang, *Journal of Applied Physics* **2018**, 124.

[2] K. F. Mak, C. H. Lui, T. F. Heinz, *Applied Physics Letters* **2010**, 97.

[3] J. Arblaster, *Platinum Metals Review* **2005**, 49, 141.

[4] E. H. Buyco, F. E. Davis, *Journal of Chemical and engineering data* **1970**, 15, 518.

[5] Z. Tong, S. Li, X. Ruan, H. Bao, *Physical review B* **2019**, 100, 144306.

[6] J. Yang, E. Ziade, A. J. Schmidt, *Review of scientific instruments* **2016**, 87.

[7] E. Sartori, *Solar energy* **2006**, 80, 1063.

[8] D. G. Cahill, *Review of scientific instruments* **2004**, 75, 5119.
